# Tuberculous Meningitis

**Published:** 1952-10

**Authors:** G. D. W. McKendrick

**Affiliations:** Deputy Resident Physician, Ham Green Hospital, Near Bristol


### TUBERCULOUS MENINGITIS

BY

G. D. W. McKENDRICK, M.A., B.M., B.CH. (OXON), M.R.C.P.
*Deputy Resident Physician, Ham Green Hospital, Near Bristol*

This is a brief review of the cases of tuberculous meningitis treated at Ham Green Hospital with streptomycin.

Since 1948, seventy-seven cases have been admitted and of these thirty-five (45 per cent) are still living. For the purpose of this review seventeen cases are excluded—eight because they have been admitted too recently and nine, the earliest cases, either because they received quite inadequate doses of streptomycin (due to an insufficient supply), or because they were transferred to other hospitals early in the disease. This leaves sixty cases which form the material for this paper. *Patients are admitted from all parts of the region and there is no selection.*

### DIAGNOSIS

The diagnosis has been confirmed in all but one case (a fatal one) by finding tubercle bacilli in the cerebro-spinal fluid. In only three cases were T.B. not found on the direct film and in two of these the organism was cultured. Thus T.B. were found in over 90 per cent of untreated cases on direct-film examination. It is essential that at least 10 c.c. of cerebro-spinal fluid should be centrifuged for two hours at 3,000 revolutions per minute, and a prolonged search carried out. It has sometimes taken three hours before T.B. have been found, but more usually they are seen in the first hour. Guinea-pig inoculation and T.B. culture are carried out routinely.

A provisional diagnosis is made on the history, signs and changes in the cerebro-spinal fluid. As is widely recognized now, the sugar level is the most important finding in the cerebro-spinal fluid and in no case has it been above 40 mgm. per cent on admission. (This does *not* include cases of miliary tuberculosis who develop meningitis whilst on treatment.) A sugar level persistently higher than 40 mgm. per cent should make the diagnosis suspect until organisms are found. However, it should be appreciated that the sugar fluctuates early in the disease and may rise considerably after starting treatment. We have recently had an early case with a sugar of 28 mgm. per cent on admission, 71 mgm. per cent the next day and 52 mgm. per cent the following day. Changes of this kind —though not of this degree—have been seen before.

Cases are classified on admission into one of three groups similar to those recommended by the Medical Research Council (1948).

*Group I* Few or no meningeal signs.

*Group II* Meningeal signs with no focal signs, or at most involvement of one cranial nerve (e.g., 3rd nerve palsy).

*Group III* Either (*I*) in coma
or (*II*) with gross focal signs—excluding a single cranial nerve palsy.

### TREATMENT

All patients received intramuscular and intrathecal streptomycin, the course of treatment depending on the patient's progress. The basic course consists of twenty-eight weeks of daily intramuscular streptomycin and six to eight weeks of daily intrathecal streptomycin. The intrathecal injections are then given on alternate days for about a month and then every third day for a further short period. There are no rest days or weeks during this period—the course being adjusted as considered necessary by the patient's progress. The intrathecal dose for children of 0–4 years is 50 mgm; of five years upwards 100 mgms. If this dose causes continual vomiting, it is reduced to 25 mgm. in infants and 50 to 75 mgm. in older children, but it is usually well tolerated.

Intramuscular streptomycin is given twice a day in a dosage of 2 gm. daily for adults, 1 gm. for adolescents and 0·5 gm. for small children. In a few recent cases the dosage has been reduced to twice-weekly injections after three months. Para-aminosalicylic acid is given in full doses. Burr holes are not made routinely, but if blocks develop streptomycin is injected into the ventricles or the cisterna magna.

Intrathecal tuberculin has been given in a few of the worst cases, but with success in only one patient who is left severely crippled with bilateral optic atrophy and some bilateral nerve deafness.

### CRITERIA OF CURE

These are freedom from signs or symptoms of the disease after a minimum of twelve months' observation, with a normal cerebro-spinal fluid, or a cerebro-spinal fluid with normal sugar and with cells and protein steadily approaching normal.

### RESULTS

Of the sixty cases, eighteen (38 per cent) are considered to be cured, thirteen are still under treatment or observation in hospital, and twenty-nine are dead. Thus at present thirty-one (50 per cent) patients are still alive but some may relapse before the completion of treatment or their observation period. (Table 1.)

TABLE 1

*Results of a series of sixty cases of tuberculous meningitis*

| Total | Dead | Surviving at present | Cured | Still under treatment or observation in hospital |
|---|---|---|---|---|
| 60 | 29 | 31 | 18 | 13 |
| 100% | 50% | 50% | 38% | 12% |

The results when cases are grouped according to their severity on admission are shown in Table 2. The last column shows that the chances of survival for an early case are twice as good as for a case in Group II and nearly four times as good as for a case in Group III. Diagnosis early in the disease is as essential for success as it is in an acute abdominal condition. A suspected case of tuberculous meningitis should be regarded as an emergency, and each day's delay in diagnosis reduces considerably the chance of survival. Nevertheless, we prefer that cases transferred to us should have received no streptomycin unless the organism has been found. The delay in transfer is only a matter of hours and treatment is started on a clinical diagnosis once a large enough specimen of cerebro-spinal fluid has been obtained for an adequate search for organisms.

TABLE 2

*Cases of tuberculous meningitis showing chances of survival related to stage of disease on admission to hospital*

| | Total | Cured | Dead | On treatment or under observation | Percentage of total cases | Percentage living at present | Percentage cured |
|---|---|---|---|---|---|---|---|
| Group I | 7 | 4 | 1 | 2 | 12 | 85 | 80 |
| Group II | 34 | 10 | 16 | 8 | 56 | 53 | 38 |
| Group III | 19 | 4 | 12 | 3 | 32 | 36 | 23 |
| Totals | 60 | 18 | 29 | 13 | 100 | — | — |

*Group I.—Few meningeal signs. Group II.—Meningeal signs but no focal signs, or single cranial nerve lesion. Group III.—In coma or with gross focal signs.*

For the period from June 1950 to November 1951 dihydro-streptomycin sulphate was used instead of streptomycin calcium chloride. Our impression, in common with other workers, was that the dihydro-streptomycin was not so effective. This is not confirmed by analysis of our figures which show no difference—38-per cent cure rate with both preparations. Our main reason for reverting to streptomycin calcium chloride, however, was the serious increase in deafness in survivors. Forty-three per cent of cases treated with dihydro-streptomycin suffered from severe bilateral nerve deafness, whereas only 6 per cent of cases treated with streptomycin calcium chloride are deaf. The only other complications in the cured cases were mild giddiness in two, and bilateral optic atrophy in one.

Reports of other workers show that the recovery rate at most centres in this country varies from 30 per cent to 50 per cent (Robertson and Gardiner, 1952, Illingsworth and Lorber, 1951, Calnan *et al.*, 1951, Cairns *et al.*, 1950). All workers agree the recovery rate is high in early cases and Illingworth (1951) says " Most children in the early stage of the disease recover and without residual defect, whereas most children in the advanced stage die, and many of the survivors are handicapped." The same may be said of adults with the added proviso that those with pronounced pulmonary disease tend to do badly.

### CONCLUSION

Our results, with 38-per cent cure, are not as good as in some series and we should like to improve them. By far the most important way of doing so is by early diagnosis—which is usually outside our control. One still sometimes hears it said that tuberculous meningitis is hardly worth treating. Any who think that should see the cures from this or any other series—happy, healthy adults and children. One ex-patient, a little girl, has recently won an open dancing competition; another girl has married and had a healthy child; another, a man of thirty, now runs an exceedingly successful caravan business. It is worth remembering that if these patients had contracted their disease five years ago, they would all be dead.

I should like to thank Dr. J. Macrae for his helpful criticisms in the preparation of this paper.

### REFERENCES

Cairns H., Smith Honor V. and Vollum R. L. (1950) *J. Amer. Med. Assoc.* **144**, 92.
Calnan, W. L., Rubie, J. and Mohun, A. F. (1951). *Brit. Med. J.* **1**, 794.
Illingsworth R. S. and Lorber J. (1951) *Lancet* **2**, 511.
Med. Research Council (1948) *Lancet* **1**, 582.
Robertson F. and Gardiner D. (1952) *Lancet* **1**, 1177.

